# Decompression of a Dentigerous Cyst in a Child With Molar-Incisor Hypomineralization: A Seven-Year Follow-Up Case Report

**DOI:** 10.7759/cureus.103697

**Published:** 2026-02-16

**Authors:** Johan Samot, Agathe Gremare

**Affiliations:** 1 Department of Oral Surgery, Bordeaux Teaching Hospital, Bordeaux, FRA; 2 Department of Odontology, Bagatelle Hospital, Bordeaux, FRA

**Keywords:** decompression surgical, molar-incisor hypomineralization, pediatric dentistry, tooth eruption, dentigerous cyst

## Abstract

Dentigerous cysts are common developmental odontogenic cysts that may compromise the eruption and morphology of developing teeth. Their management of children increasingly favors conservative approaches, particularly when tooth preservation is possible. Molar-incisor hypomineralization (MIH) introduces additional clinical considerations due to enamel fragility, which may affect patient comfort and complicate postoperative hygiene.

An eight-year-old boy with MIH presented with a dentigerous cyst associated with the unerupted mandibular right second molar (tooth 47), incidentally identified on a panoramic radiograph. Cone-beam computed tomography confirmed a well-defined unilocular cyst enveloping the crown of the tooth. Decompression was performed to preserve the developing tooth. Progressive radiographic improvement was observed over the following months, with gradual repositioning and eruption of the tooth into functional occlusion despite altered root development. No recurrence was detected during a seven-year follow-up. Management of MIH-related fragility included fluoride application, enhanced oral hygiene, and restorative treatment.

This case highlights the feasibility of tooth preservation despite altered root development and MIH-related enamel fragility. Early diagnosis, detailed imaging, and structured follow-up are essential for achieving favorable outcomes.

## Introduction

Molar-incisor hypomineralization (MIH) is a developmental enamel defect primarily affecting permanent first molars and, to a lesser extent, permanent incisors [1]. Hypomineralized enamel is more porous and brittle, leading to post-eruptive breakdown and an increased susceptibility to dental caries. Management of the resulting tissue loss is often challenging, as MIH is frequently associated with dental hypersensitivity and reduced anesthetic efficacy [2]. Consequently, affected children tend to present with significantly greater treatment needs, particularly in severe cases. These repeated dental visits may also provide an opportunity to detect other oral pathologies incidentally, requiring prompt management.

Dentigerous cysts are developmental odontogenic cysts associated with the crowns of unerupted teeth and are among the most common jaw cysts in children. While enucleation has historically been the standard treatment, conservative approaches such as marsupialization and decompression are increasingly favored to preserve developing teeth and minimize surgical morbidity [3]. In children with MIH, enamel fragility and hypersensitivity of adjacent teeth may complicate postoperative care, particularly with respect to oral hygiene maintenance, and may therefore influence treatment decisions when balancing conservative and more radical approaches.

This report describes the conservative management of a dentigerous cyst in a child with MIH, emphasizing the clinical feasibility of tooth preservation and the importance of long-term follow-up.

## Case presentation

An eight-year-old boy was referred to the pediatric dentistry unit for evaluation of suspected MIH. As part of routine follow-up, a panoramic radiograph was obtained, revealing a well-defined unilocular radiolucency surrounding the crown of the unerupted mandibular right second molar (tooth 47), which was displaced toward the basal bone.

**Figure 1:**
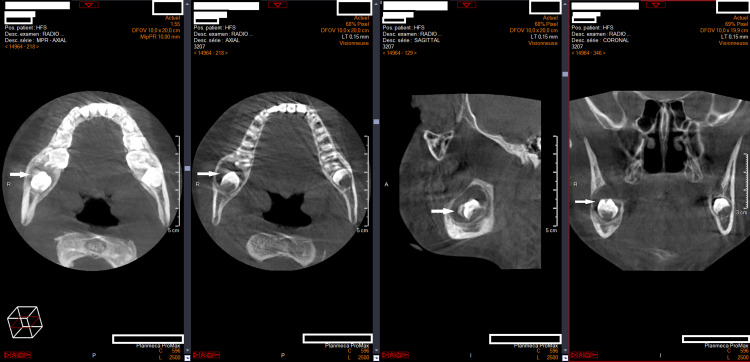
CBCT of a radiolucent lesion around the unerupted right mandibular second molar Axial, sagittal, and coronal CBCT views showing a well-circumscribed unilocular radiolucency with a thin sclerotic border surrounding the crown of the unerupted right mandibular second molar. White arrows indicate the radiolucent lesion enveloping the crown of the developing tooth germ. CBCT: cone-beam computed tomography

The patient was asymptomatic, with no clinical swelling or signs of infection. Cone-beam computed tomography (CBCT) was performed to further assess the lesion and its relationship to adjacent anatomical structures. CBCT imaging (Figure 1) demonstrated a unilocular cystic lesion with well-corticated borders enveloping the crown of tooth 47, without evidence of cortical perforation, findings consistent with a dentigerous cyst.

The adjacent mandibular right first molar (tooth 46) exhibited typical MIH features, including demarcated enamel opacities and post-eruptive enamel breakdown.

**Figure 2:**
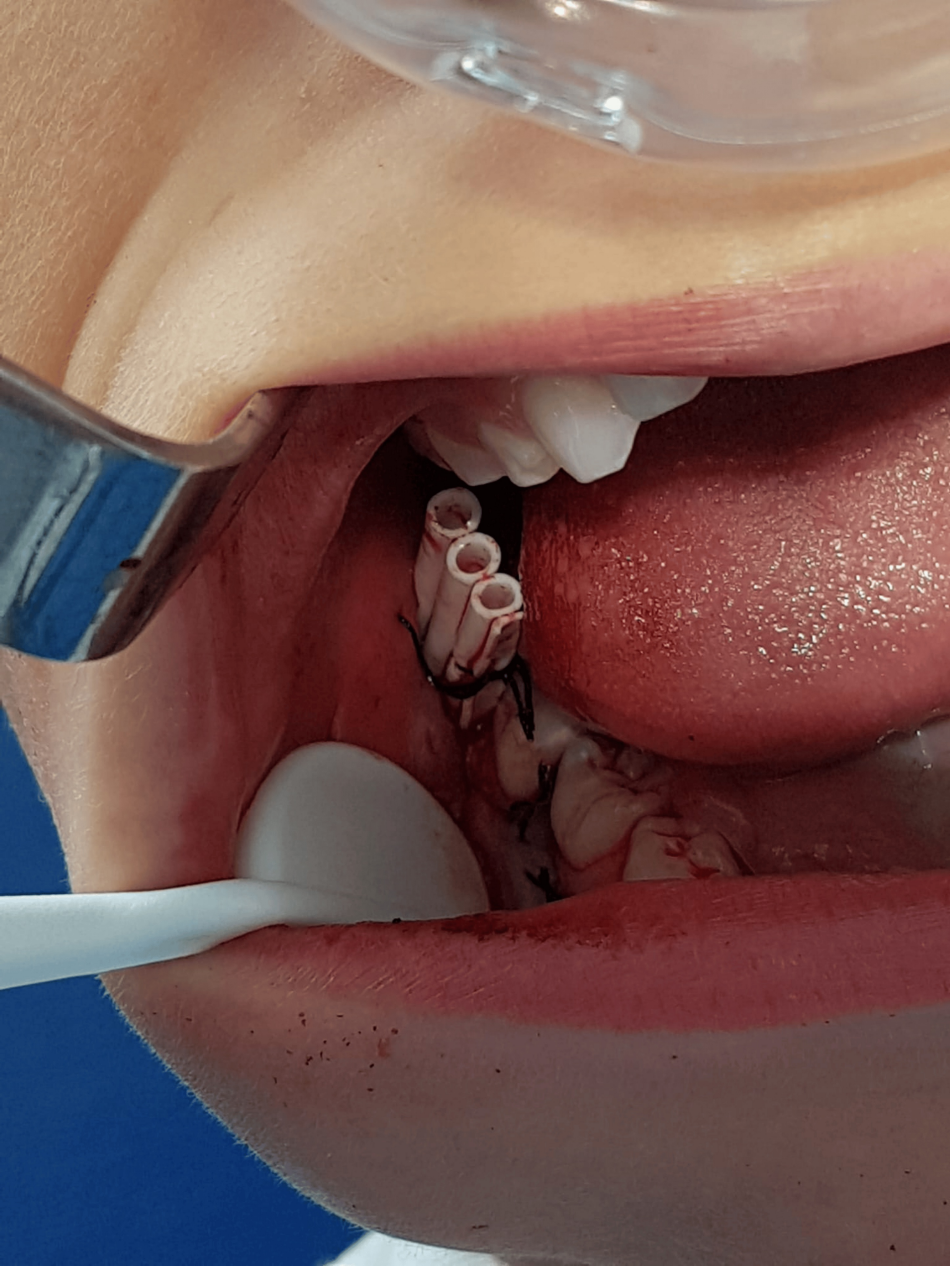
Intraoral view after decompression surgery Intraoral photograph showing the buccally sutured surgical drain following decompression (EMONO sedation mask visible). EMONO: equimolar mixture of oxygen and nitrous oxide

Given the patient’s age and the desire to preserve the developing second molar, a conservative decompression procedure was selected. Surgery was performed under local anesthesia with adjunctive analgesia and anxiolysis using an equimolar mixture of oxygen and nitrous oxide (EMONO). A small bony window was created, and a surgical drain was placed to maintain continuous decompression of the cystic cavity (Figure 2).

**Figure 3:**
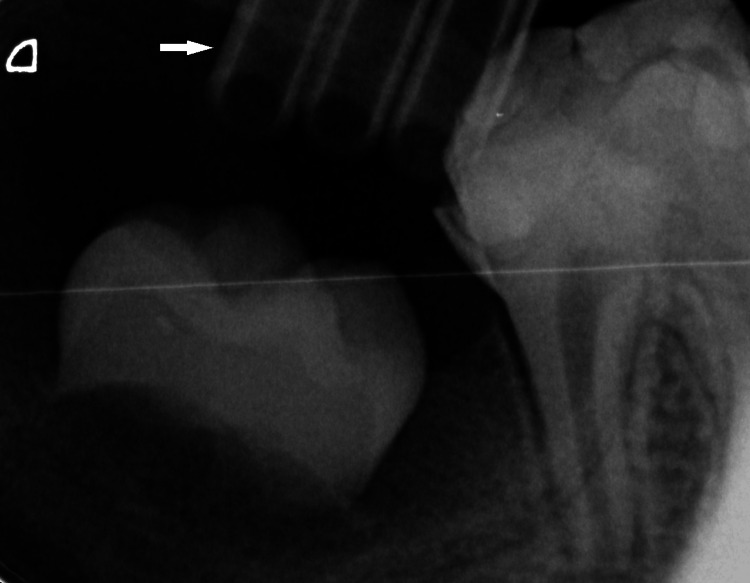
Radiographic assessment of drain positioning Periapical radiograph illustrating the position of the surgical drain relative to the radiolucent lesion and the unerupted tooth germ. The white arrow indicates the surgical drain.

Proper positioning of the drain was confirmed radiographically (Figure 3).

Postoperative care included analgesic medication and alcohol-free 0.12% chlorhexidine mouthwash. Additionally, twice-daily irrigation with normal saline was performed through the surgical drain for one month.

Postoperative recovery was uneventful. Histopathological examination of the cystic lining revealed a fibrous wall lined with non-keratinized stratified squamous epithelium, confirming the diagnosis of a dentigerous cyst.

**Figure 4:**
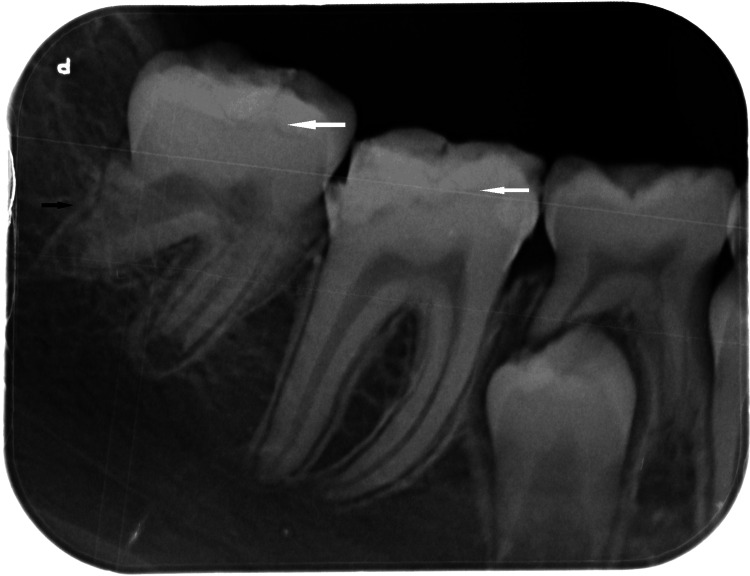
Long-term radiographic outcome showing eruption of the mandibular right second molar despite altered root development Periapical radiograph showing hypomineralized enamel affecting teeth 46 and 47 and an abnormal distal root morphology of tooth 47 following conservative management. White arrows indicate the enamel defects, while the black arrow highlights the divergent distal root of tooth 47.

The patient was followed closely with regular clinical and radiographic evaluations. Progressive reduction of the cystic cavity and gradual repositioning of tooth 47 were observed. Although the involved tooth's root development was altered, with shortened and divergent roots, spontaneous eruption occurred, and the tooth achieved functional occlusion (Figure 4).

No signs of recurrence were detected during the seven-year follow-up period. Due to the structural compromise associated with MIH, tooth 46 was restored with a full-coverage indirect restoration to ensure long-term structural integrity and function.

## Discussion

Dentigerous cysts are developmental odontogenic cysts most frequently associated with the crowns of unerupted permanent teeth, particularly in the mandibular molar region. The treatment of these lesions has evolved from systematic surgical removal toward more conservative approaches, especially in pediatric patients. While enucleation remains a standard treatment modality [4-6], more conservative techniques, such as marsupialization [7] or decompression [8], have gained acceptance. These procedures allow for a gradual reduction of cystic volume while preserving the involved tooth and surrounding structures. Current management trends even favor tooth preservation in cases complicated by cyst infection [9].

In the present case, decompression proved effective in promoting spontaneous repositioning and eruption of the displaced mandibular second molar (tooth 47), avoiding premature extraction or extensive surgical intervention. Although some authors recommend a two-stage approach with subsequent enucleation to prevent recurrence [10], increasing evidence suggests that decompression alone may be adequate when long-term monitoring is ensured [11].

The success of this conservative approach depended on early diagnosis, appropriate imaging, and strict postoperative monitoring. CBCT was used for preoperative assessment to accurately evaluate the cyst’s dimensions and its relationship with adjacent anatomical structures, thereby facilitating surgical planning. Postoperative follow-up was performed using panoramic and periapical radiographs to monitor bone regeneration and tooth eruption. Although CBCT enables three-dimensional visualization of intraosseous lesions, its use is not always necessary, as evidence suggests that it does not improve clinical outcomes [12]. This case also illustrates the added complexity introduced by MIH, which can influence both the surgical and postoperative course. MIH-affected teeth often display structural fragility, hypersensitivity, and reduced anesthetic efficacy. In our patient, however, no intraoperative anesthetic difficulty was encountered. The anticipatory use of EMONO for analgesia and anxiolysis, as recommended by Prud’homme et al. [13], may have contributed to the child’s comfort and cooperation during the procedure.

The fragility of MIH-affected enamel also guided postoperative management. Although MIH can complicate oral hygiene, the strong involvement of the parents, frequent follow-up appointments, and regular fluoride varnish applications, whose effectiveness in preventing dental caries in permanent teeth has been well documented [14], ensured sufficient hygiene around the decompression drain. The placement of glass ionomer sealants on occlusal fissures, materials with demonstrated remineralizing potential for carious lesions [15], likely supported the maintenance of pulpal vitality and helped prevent new lesions over time.

The root morphology of tooth 47, characterized by shortened and divergent roots, was most likely the consequence of mechanical displacement caused by the lesion.

Despite this impaired root development, the tooth erupted into functional occlusion, supporting the hypothesis that immature teeth enclosed within a cyst can retain their eruptive potential following decompression [16]. This observation aligns with several reports describing tooth eruption even in the absence of fully formed roots and highlighting the key regulatory role of the dental follicle [17]. Such findings further support a conservative approach to the management of dentigerous cysts when histopathological examination confirms their benign nature. Nevertheless, long-term follow-up remains essential to ensure the absence of further complications, including recurrence or, more rarely, malignant transformation.

## Conclusions

This case demonstrates that decompression is a reliable and minimally invasive treatment option for dentigerous cysts in pediatric patients, even in the presence of complicating factors such as MIH. With early diagnosis, appropriate imaging, and structured long-term follow-up, tooth preservation and functional eruption can be achieved despite altered root development. Clinicians should consider conservative management strategies as a first-line approach in similar pediatric cases. Although based on a single case, these findings provide supportive evidence for this treatment strategy and warrant further investigation using standardized, objective follow-up measures in larger patient cohorts.
